# Case Report: A successful case of allogeneic stem cell transplantation for pediatric XMEN characterized by neutropenia

**DOI:** 10.3389/fimmu.2026.1687549

**Published:** 2026-01-15

**Authors:** Jieyu Tian, Jie Zheng, Maoquan Qin, Huawei Mao, Guanghua Zhu

**Affiliations:** 1 Hematology Center, Beijing Key Laboratory of Pediatric Hematology Oncology, National Key Discipline of Pediatrics (Capital Medical University), Key Laboratory of Major Diseases in Children, Ministry of Education, Beijing Children’s Hospital, Capital Medical University, National Center for Children’s Health, Beijing, China; 2 Department of Immunology, Beijing Children’s Hospital, Capital Medical University, National Center for Children’s Health, Beijing, China

**Keywords:** HSCT = hematopoietic stem cell transplant, IEI, inborn errors of immunity, MAGT1 deletion, neutropenia, case report

## Abstract

XMEN disease (X-linked immunodeficiency with magnesium defect, EBV infection, and neoplasia) is a rare Inborn Error of Immunity (IEI)characterized by impaired magnesium ion transport due to mutations in the *MAGT1* gene, which subsequently affects immune cell function. Timely diagnosis and prompt intervention are essential for improving patient outcomes. Allogeneic hematopoietic stem cell transplantation (HSCT) offers a potential therapeutic approach to restore *MAGT1* function. We report an infant with XMEN who acquired a novel mutation in the *MAGT1* gene, presenting recurrent severe skin infections and neutropenia after 6 months of age, which was effectively managed following aggressive anti-infective treatment and HSCT.

## Introduction

XMEN disease is a congenital IEI resulting from the loss of function of the gene encoding magnesium transporter 1. This condition is typically characterized by Epstein-Barr virus (EBV) infection, chronic sinopulmonary infections, and an increased susceptibility to EBV-associated lymphomas (1, 2). *MAGT1* is a conserved magnesium-specific transporter consisting of a gene of approximately 70 kb in length with 10 exons, which encodes a protein composed of 335 amino acids. The gene exhibits broad expression across all mammalian cells, with particularly high levels in the hematopoietic lineage. Mutations of this gene lead to reduced expression of NKG2D, an essential activating receptor for anti-EBV cytotoxicity. It is widely accepted that the diminished expression of CD8+ T-cells and natural killer (NK) cells in patients with XMEN represents a novel mechanism contributing to the increased susceptibility of children to infections, particularly those with EBV. The disease can manifest at any age, from early childhood to adulthood, due to its association with the X chromosome, all reported individuals are male. HSCT is a critical treatment option for some patients, but complications following transplantation present significant challenges (3). This report details a child with XMEN who carried a partial deletion of a *MAGT1* exon successfully received allogeneic hematopoietic stem cell transplantation at our institution.

## Case presentation

**Figure 1:**
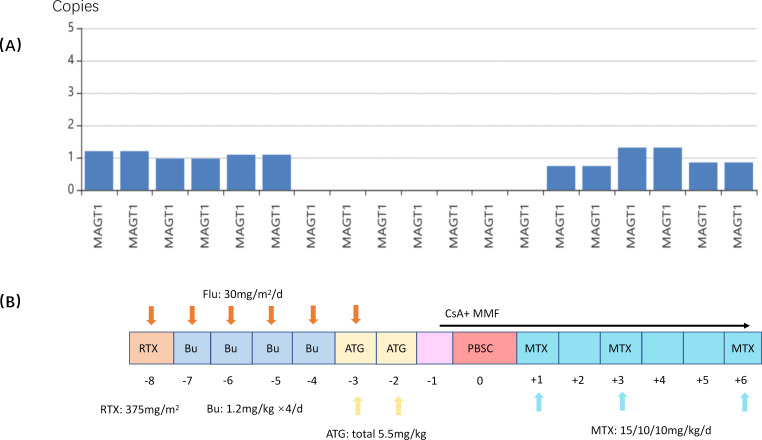
**(A)** The MAGT1 gene is encoded by 10 exons, and Whole-Exome Sequencing shows the patient lose the segment between exons 4-7. **(B)** Pretreatment regimen: fludarabine (Flu) 180 mg/m2, Bu 4.8 mg/Kg/d*4d; rituximab (RTX) 375mg/m^2^ for prevention of post-transplant lymphoproliferative disorders; ATG/CsA/MMF/MTX for prevention of GVHD.

The child demonstrated normal fetal development but subsequently developed *Staphylococcus epidermidis* septicaemia and pneumonia during the neonatal period. He was subsequently discharged following anti-infective therapy. At the age of six months, he was identified with neutropenia with a count of 0.97*10^9/L, accompanied by lip ulcers and hematuria. Immunological evaluation revealed significant hypogammaglobulinemia, characterized by decreased levels of IgA at 0.2 g/L (reference range: 0.3–1.4 g/L), IgM at 0.22 g/L (reference range: 0.3–1.0 g/L), and IgG at 4.66 g/L (reference range: 3.0–10.0 g/L). Clinically, the patient presented with recurrent, severe bacterial and viral infections affecting the urinary tract, lungs, and soft tissues, including episodes of sepsis. Hematological analysis showed severe neutropenia (~2% of total leukocytes) despite a normal or elevated white blood cell count. Hematological parameters showed that hemoglobin and platelet counts remained consistently within normal limits (**Table 1**). Initially, each infectious episode responded favorably to antibiotic therapy. However, as the patient aged, the infections became progressively refractory to treatment, even with the administration of broad-spectrum or escalated antibiotic regimens. A partial deletion of the *MAGT1* gene (exons 4-7) was identified by WES (Whole Exome Sequencing) analysis at 12 months of age, establishing it as the underlying etiology (**Figure 1A**). The real-time quantitative PCR (qPCR) method was performed to confirm the genetic mutation, and his mother was identified as carrier.

**Table 1:** Clinical phenotype of XMEN patient.

Characteristics	Details
Age	12 months
Mutation Gemomic	*MAGT1* EXON4-7 deletion
Urinary infection	*Enterococcus faecium*
Pneumonia	*Streptococcus pneumoniae/ Parainfluenza virus/Cytomegalovirus*
Skin and soft tissue infections	*Pseudomonas aeruginosa/ Candida tropicalis*
EBV	Negative
Cancer	Negative
Peripheral blood cells	Outcomes
T cells	71.6%
CD4 T cells	24.7%
CD8 T cells	39.3%
CD4:CD8	0.63
B cells	19.6%
Eosinophils	0.1%
Neutrophils	3%
Monocytes	5.6%
Lymphocyte subset	(Normal range for age)
CD3+CD4+	17.5%(24.08-42.52%)
CD45+	70%(46.14-84.4%)
CD45-	30%(14.88%-54.64%)
CD3+CD8+	17.6(19-32.51%)
CD4-CD8-	3.4%(0.37%-1.8%)
B cell	50.2%(13.23-26.39%)
NK cell	9%(7.21-20.9%)
T Lymphocyte subset	Outcomes(Normal range for age)
CD4+IL-4+	0.3%(0.3-2%)
CD4+INF-γ+	3.95%(6.5-28%)
CD25+FOXP3+	5.33%(4.1-9.4%)
IL-17+	0.86%(0.2-2.4%)
Functional tests	Outcomes
TRECs	Normal
KRECs	Normal
CD107a Degranulation Assay	Outcomes(Normal range for age)
NK cell	10.18%(>10%)
CTL cell	4.8%(>2.8%)
NK Cell Activity Assay	12.64%(>15.11%)
Immunoglobulin levels	Outcomes(Normal range for age)
IgG, g/L	4.66(3-10)
IgA, g/L	0.2(0.3-1.4)
IgM g/L	0.22(0.3-1.0)

He was admitted to our center with an infection of the eyelids and perianal skin at 13-months-old. Despite treatment with subcutaneous recombinant human granulocyte colony-stimulating factor (rhG-CSF), the patient’s neutrophil count peaked at only 0.8×10^9^/L. A subsequent bone marrow analysis revealed hypoproliferative granulopoiesis. Although the patient’s fever subsided with conservative management and systemic antibiotics, the soft tissue infection progressed, and new abscesses developed on the thighs. Cultures from the lesions grew *Pseudomonas aeruginosa* and *Candida tropicalis*. Given the definitive genomic diagnosis, early-onset severe infections refractory to conventional therapy, the patient met the criteria for hematopoietic stem cell transplantation (HSCT). Prior to HSCT, the patient received two surgical incision and drainage procedures to control the abscesses. Following the resolution of skin infections, the patient proceeded with an allogeneic HSCT from a 10/10 HLA-matched unrelated donor. The pretreatment regimen included fludarabine (180 mg/m^2^), busulfan (1.2 mg/kg/dose every 6 hours for a total of 4 days, the dose was adjusted based on the measured concentrations, yielding a predicted cumulative AUC of 84.51 mg*h/L) and ATG (antithymocyte globulin 5.5mg/kg). Prophylaxis for acute graft-versus-host disease (aGVHD) consisted of mycophenolate mofetil (MMF), cyclosporine (CsA), and methotrexate (MTX) (**Figure 1B**). The graft contained a total nucleated cell (TNC) count of 13.06×10^8^/kg, a CD34+ cell count of 4.3×10^6^/kg, and a CD3+ of 5.6 × 10^8^/kg.

**Figure 2:**
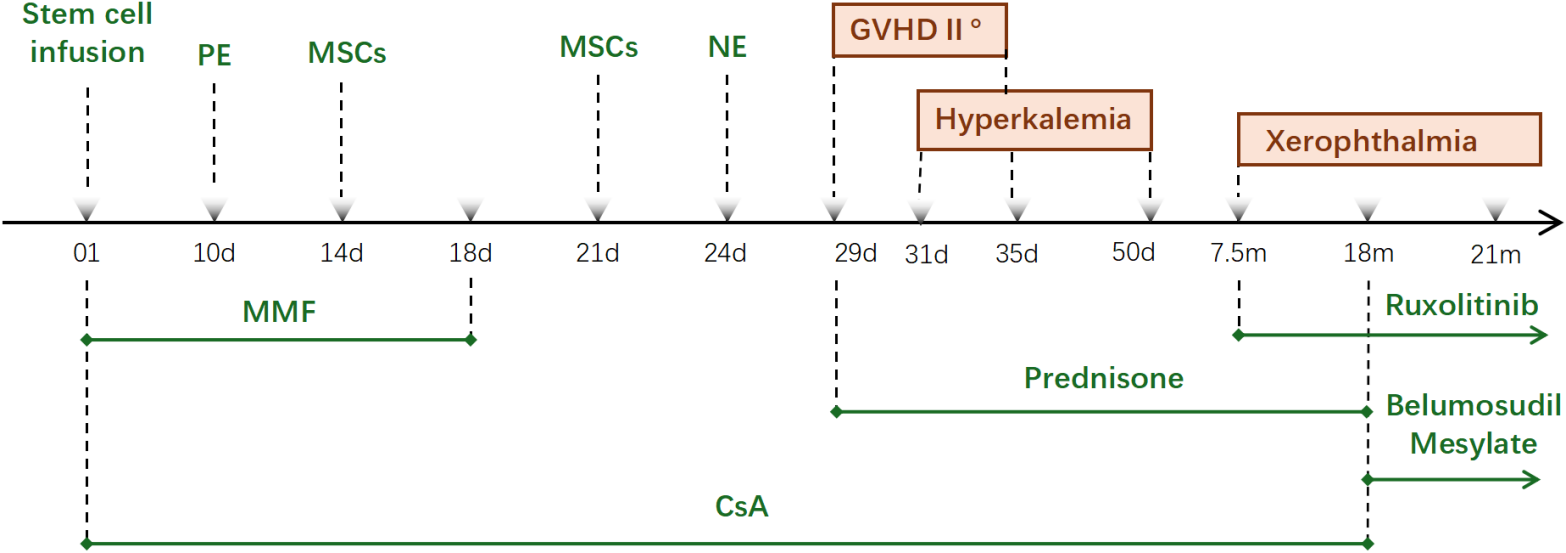
Therapeutic timeline. PE : Platelet engraftment; NE : neutrophils engraftment; MSCs : mesenchymal stromal cells.

During the transplantation period, the patient developed a transient low-grade fever, blood cultures grew Staphylococcus epidermidis. The fever resolved promptly with empiric antibiotic therapy. Platelet engraftment occurred on day 10, and neutrophils engraftment on day 24. To promote neutrophil engraftment, mesenchymal stromal cells (MSCs 1 × 10^7^/kg) were infused on day 14 and day 21 post-HSCT. On day 29, the patient developed watery diarrhea and was diagnosed with Grade II graft-versus-host disease (GVHD) on the basis of the MAGIC criteria. Treatment was initiated with prednisone (2 mg/kg/day)and oral budesonide. On day 34, the patient developed thrombocytopenia, presumed to be immune-mediated, which improved with the administration of hormones and intravenous immunoglobulin. Regarding immunosuppression management, mycophenolate mofetil (MMF) was discontinued on day 18. Oral cyclosporine was continued for the management of chronic graft-versus-host disease (cGVHD) until 18 months post-transplant, with clinical manifestations including dry eyes and skin involvement. Ruxolitinib and belumosudil were also administered as part of the cGVHD treatment regimen (**Figure 2**).

**Figure 3:**
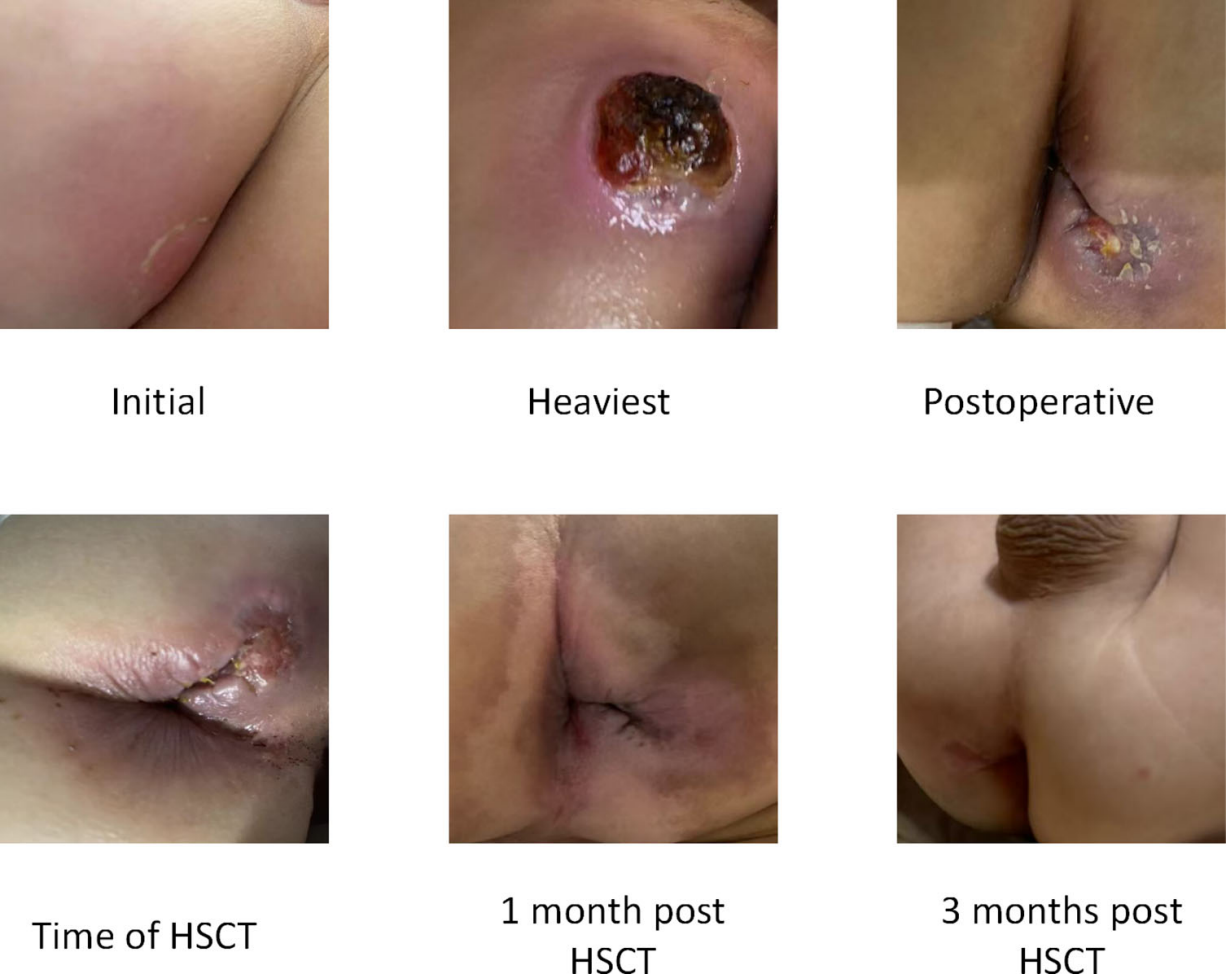
Skin infection was severe and improved rapidly after the HSCT.

**Figure 4:**
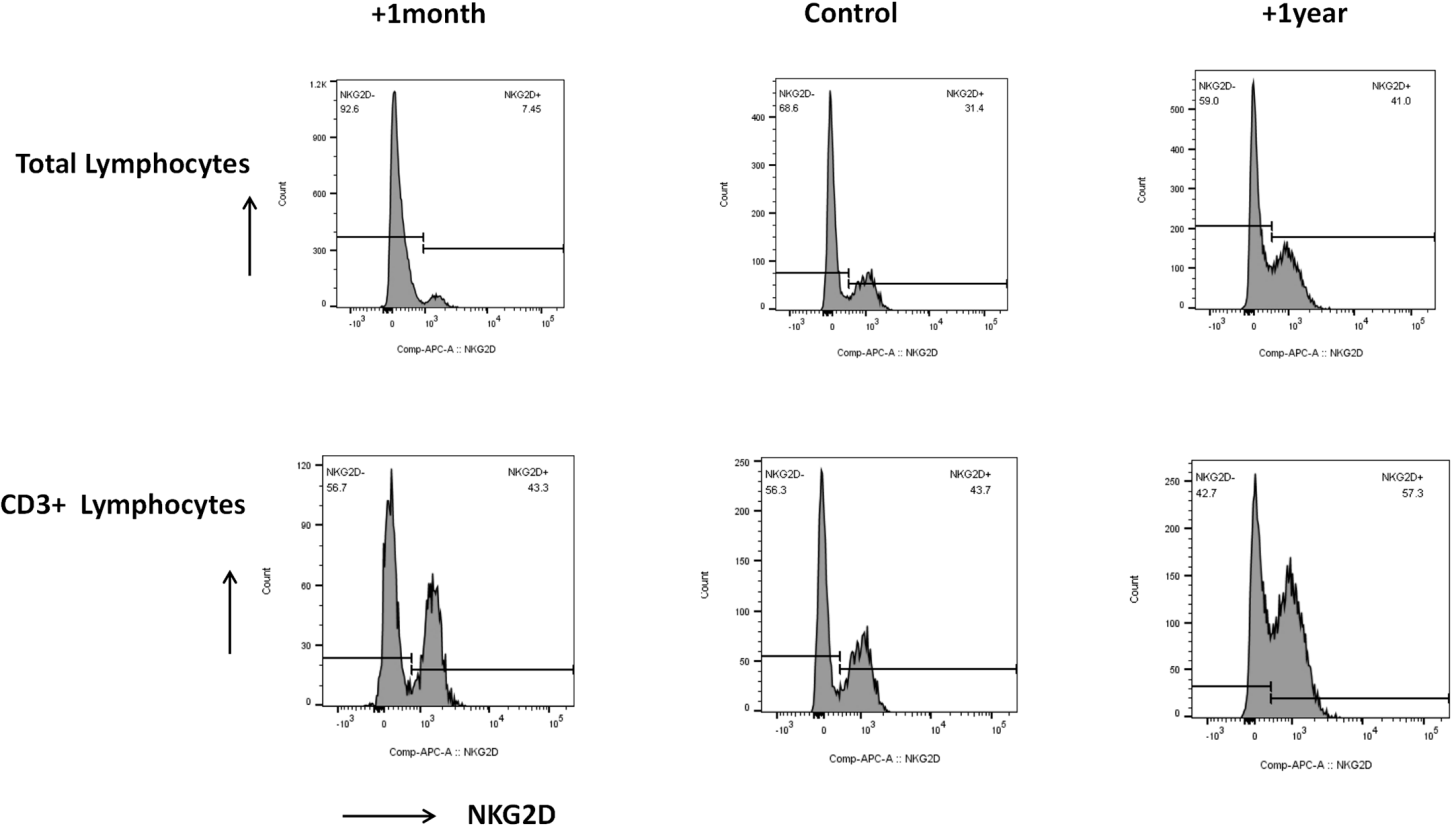
NKG2D expression in total lymphocytes was 7.45% and 41% after 1 month and 1year HSCT respectively, suggesting a low initial expression value of NKG2D compared with 31.4% in normal child of the same age; while 43.3% and 57.3% in CD3+ lymphocytes at 1 month and 1 year post-transplantation, respectively, suggesting no significant change compared with the 43.7% expression in normal children.

Monitoring has shown sustained complete donor engraftment. The previously infected foci in the perianal area and thighs showed gradual healing (**Figure 3**), with no new refractory infections since discharge. The patient’s NKG2D expression, which was significantly low at one month post-transplantation, had normalized by the one-year follow-up when compared to age-matched healthy controls (**Figure 4**).

## Discussion

The clinical presentation of this patient was atypical for X-linked immunodeficiency with magnesium defect, EBV infection, and neoplasia (XMEN) syndrome. Key deviations from the classic phenotype included sustained negativity for Epstein-Barr virus (EBV) and preserved cytotoxic T lymphocyte (CTL) and NK cell counts. However, the diagnosis was firmly supported by the significantly low expression of NKG2D on total lymphocytes. Further flow cytometric analysis revealed that NKG2D expression was preserved on CD3+ T-cells, pinpointing the deficiency to other lymphocyte subsets, particularly NK cells (**Figure 3**). A significant feature of our case was persistent, severe neutropenia (absolute neutrophil count consistently around 0.5×10^9^/L during the first year of life), which is not a hallmark of XMEN. Bone marrow aspiration confirmed a hypoproliferative granulocyte lineage. This finding contrasts with reports of transient neutropenia in some XMEN patients and differs from the milder neutropenia reported in a patient with a 10-base-pair deletion (4). We are the first to report a large, multi-exon deletion (exons 4-7) in the *MAGT1* gene. Other reports have suggested that *MATG1* might positively regulate the progression of breast cancer, highlighting its pleiotropic effects (5). An intriguing possibility arises that this specific genomic alteration may disrupt a previously unrecognized regulatory function of *MAGT1* in neutrophil proliferation and differentiation, although this remains to be supported by further investigation.

XMEN syndrome in adults often has a more indolent course with manageable infections, where conservative treatment may be appropriate. A systematic review indicated a correlation between EBV infection and an unfavorable prognosis in this disorder (6). Magnesium supplementation has been reported to enhance NKG2D expression (7), its clinical efficacy in preventing infections or malignancy remains controversial. While gene therapy demonstrates curative potential in preclinical and clinical reports, it is not yet widely accessible (8). Thus, our severely affected infant opted for hematopoietic stem cell transplantation(HSCT).

The decision for early transplantation was supported by extensive evidence from other primary immunodeficiency diseases (PIDs). Multicenter studies on Wiskott-Aldrich syndrome (WAS), leukocyte adhesion defects (LAD), and chronic granulomatous disease (CGD) have consistently shown that performing HSCT in infancy or early childhood, prior to the onset of severe infections and irreversible end-organ damage, is associated with significantly higher overall and event-free survival rates (9–11). Although HSCT for XMEN can be challenged by high rates of transplant-related complications, potentially exacerbated by endothelial damage from prior infections, the potential benefits of early intervention in our patient outweighed these risks.

The TCRαβ depletion technique holds promise in pediatric PID transplantation, largely because it correlates with lower incidence of GVHD and more rapid engraftment (12). Graft rejection, though a recognized risk, has been mitigated to acceptable levels through protocol refinements like adjunctive bone marrow or CD45RA-negative cell infusion (13, 14). However, the high cost significantly limits its broader application in many countries. In the present case, the child was an only child and conceived via donor sperm IVF, making the biological father unavailable. Therefore, a matched unrelated donor (MUD) was a superior choice compared to using his phenotypically normal carrier mother as the donor.

The choice of conditioning regimen was critical. Most of the studies concluded that busulfan(Bu) combined with fludarabine(Flu) was less toxic and had a lower incidence of late adverse events than the busulfan cyclophosphamide pretreatment regimen (15). A study suggests treosulfan-based increased the rate of engraftment failure and the possibility of lower myeloid donor chimerism compared to busulfan (9). However, the concentration of Busulfan varies greatly among children, and insufficient exposure may lead to transplant failure. Strict concentration monitoring is required, and attention should be paid to the risk of liver damage and Veno-occlusive disease (VOD) caused by high concentrations. And when using FLU+BU, sufficient drug exposure must be strictly ensured. Based on our institutional experience and literature supporting its favorable toxicity profile, a myeloablative conditioning (MAC) regimen comprising Busulfan, Fludarabine, and anti-thymocyte globulin (Bu/Flu/ATG) was administered. Rigorous therapeutic drug monitoring for busulfan was employed to ensure adequate exposure while minimizing toxicity risks like VOD. A crucial preparatory step involved aggressive infection control, including surgical debridement of refractory skin infections, to optimize the patient’s condition for HSCT. The post-transplantation course was successful, with no severe infections or GVHD. A notable and previously unreported complication was transient, recurrent hyperkalemia (peak 6.47 mmol/L) observed from day 32, which resolved with symptomatic management. We speculate this may be related to altered magnesium-potassium ion transport dynamics following donor cell engraftment. Encouragingly, our patient did not exhibit the severe bleeding diatheses that have been reported in some XMEN patients post-HSCT (16).

We report a case of infantile-onset XMEN syndrome caused by a novel, multi-exon deletion in the *MAGT*1 gene. This genomic defect was associated with an atypical phenotype of severe, persistent neutropenia, suggesting a potential new role for *MAGT1* in granulopoiesis. Our experience demonstrates that early allogeneic HSCT, using a myeloablative Bu/Flu/ATG conditioning regimen in an experienced center, can be a safe and curative approach for severely affected children. This strategy facilitated stable donor engraftment, resolved refractory infections, highlighting the importance of early, definitive intervention in this rare and complex IEI.

## Conclusion

This report presents a novel case of a child with XMEN syndrome characterized by neutropenia as the primary feature of infantile onset, accompanied by severe infections. The patient achieved positive outcomes following an intensive anti-infective strategy and subsequent allogeneic hematopoietic stem cell transplantation.

## Data Availability

The raw data supporting the conclusions of this article will be made available by the authors, without undue reservation.
